# Supplementary material for: Improving cassava bacterial blight resistance by editing the epigenome
Source: Nat Commun. 2023 Jan 5;14:85. doi: 10.1038/s41467-022-35675-7 (PMC9816117; doi:10.1038/s41467-022-35675-7)
Supplement: Supplementary file 2 — Reporting Summary [file 41467_2022_35675_MOESM2_ESM.pdf]

nature portfolio

Corresponding author(s): *Double-blind peer review submissions: write DBPR and your manuscript number here instead of author names.*

Last updated by author(s): *YYYY-MM-DD*

Reporting Summary

Nature Portfolio wishes to improve the reproducibility of the work that we publish. This form provides structure for consistency and transparency in reporting. For further information on Nature Portfolio policies, see our Editorial Policies and the Editorial Policy Checklist.

Statistics

For all statistical analyses, confirm that the following items are present in the figure legend, table legend, main text, or Methods section.

n/a	Confirmed	
☐	☒	The exact sample size (*n*) for each experimental group/condition, given as a discrete number and unit of measurement
☐	☒	A statement on whether measurements were taken from distinct samples or whether the same sample was measured repeatedly
☐	☒	The statistical test(s) used AND whether they are one- or two-sided<br>*Only common tests should be described solely by name; describe more complex techniques in the Methods section.*
☐	☒	A description of all covariates tested
☐	☒	A description of any assumptions or corrections, such as tests of normality and adjustment for multiple comparisons
☐	☒	A full description of the statistical parameters including central tendency (e.g. means) or other basic estimates (e.g. regression coefficient) AND variation (e.g. standard deviation) or associated estimates of uncertainty (e.g. confidence intervals)
☐	☒	For null hypothesis testing, the test statistic (e.g. *F*, *t*, *r*) with confidence intervals, effect sizes, degrees of freedom and *P* value noted<br>*Give P values as exact values whenever suitable.*
☒	☐	For Bayesian analysis, information on the choice of priors and Markov chain Monte Carlo settings
☒	☐	For hierarchical and complex designs, identification of the appropriate level for tests and full reporting of outcomes
☒	☐	Estimates of effect sizes (e.g. Cohen's *d*, Pearson's *r*), indicating how they were calculated

Our web collection on statistics for biologists contains articles on many of the points above.

Software and code

Policy information about availability of computer code

Data collection	No software was used for data collection,
Data analysis	For watersoaking analysis, images were first gray-corrected to normalize brightness using a publicly available tool (https://github.com/jberry47/ddpsc_phenotypercv) then analyzed with FIGI (version 2.1.0/1.53c).). A ChemiDoc XRS+ System equipped with Image Lab 6.1 software (Bio-Rad) was used for imaging and quantifying westerns. For whole-genome BS-seq, BSMAP (v2.90) was used for mapping, duplicated reads were removed with SAMtools (v1.3.1) and methylation track files were visualized with Integrative Genomics Viewer (IGV, v3.0). For off-target sites was predicted by Cas-OFFinder and sequence consensus analysis of potential off-target sites was performed with WebLogo (v3.6.0).

For manuscripts utilizing custom algorithms or software that are central to the research but not yet described in published literature, software must be made available to editors and reviewers. We strongly encourage code deposition in a community repository (e.g. GitHub). See the Nature Portfolio guidelines for submitting code & software for further information.

Data

Policy information about availability of data

All manuscripts must include a data availability statement. This statement should provide the following information, where applicable:

- Accession codes, unique identifiers, or web links for publicly available datasets
- A description of any restrictions on data availability
- For clinical datasets or third party data, please ensure that the statement adheres to our policy

All amplicon-based bisulfite sequencing data, western blots, images used for imaged-based analysis of water-soaking disease symptoms, and R code for generating plots are available in a FigShare repository (DOI: 10.6084/m9.figshare.16887934). Whole genome bisulfite sequencing data is available at GEO:
GSM5667182 - WGBS data of wild type TME419
GSM5667183 - WGBS data of DMS3-ZF line 133
GSM5667184 - WGBS data of DMS3-ZF line 204

Human research participants

Policy information about studies involving human research participants and Sex and Gender in Research.

Reporting on sex and gender	Does not apply
Population characteristics	Does not apply
Recruitment	Does not apply
Ethics oversight	Does not apply

Note that full information on the approval of the study protocol must also be provided in the manuscript.

Field-specific reporting

Please select the one below that is the best fit for your research. If you are not sure, read the appropriate sections before making your selection.

☒ Life sciences ☐ Behavioural & social sciences ☐ Ecological, evolutionary & environmental sciences

For a reference copy of the document with all sections, see nature.com/documents/nr-reporting-summary-flat.pdf

Life sciences study design

All studies must disclose on these points even when the disclosure is negative.

Sample size	Experiments quantifying disease resistance in cassava generated from tissue culture are labor and resource-intensive, so plant availability was limited. To this data with adequate replication, multiple rounds of cassava plants were brought out of tissue culture and each set was used for as many individual experimental sets as possible. The paper is written with these limitations in mind, and we are transparent about which sets of plants were used for specific experiments. All experiments were preformed in triplicate.
Data exclusions	One round of plants was excluded from analysis because controls did not behave correctly (WT plants treated with Xam did not show disease symptoms). Otherwise, all samples were used.
Replication	We report all experimental reps performed where controls gave the expected outcome. The wet-lab experiments shown were performed over the course of five years. The field work is ongoing, but the data presented here was collected over the course of 6 months.
Randomization	Plants were randomized during growth, before and after CBB treatment.
Blinding	Researchers were not blind during data collection or analysis due to practical limitations and insuring replication number (see note about plant availability above).

Reporting for specific materials, systems and methods

We require information from authors about some types of materials, experimental systems and methods used in many studies. Here, indicate whether each material, system or method listed is relevant to your study. If you are not sure if a list item applies to your research, read the appropriate section before selecting a response.

Materials & experimental systems

n/a	Involved in the study	
☐	☒	Antibodies
☒	☐	Eukaryotic cell lines
☒	☐	Palaeontology and archaeology
☒	☐	Animals and other organisms
☒	☐	Clinical data
☒	☐	Dual use research of concern

Methods

n/a	Involved in the study	
☒	☐	ChIP-seq
☒	☐	Flow cytometry
☒	☐	MRI-based neuroimaging

Antibodies

Antibodies used	For western blotting, monoclonal ANTI-FLAG® M2-Peroxidase (HRP) antibody (Catalog #A8592, Sigma) and and anti-His antibody (His-Tag (27E8) Mouse mAb (HRP Conjugate), Catalog #9991, Cell Signaling Technology) were used.
Validation	Validation was based on the size of observed bands detected and comparison to controls (i. e. samples with and without expression).
